# NA Proteins of Influenza A Viruses H1N1/2009, H5N1, and H9N2 Show Differential Effects on Infection Initiation, Virus Release, and Cell-Cell Fusion

**DOI:** 10.1371/journal.pone.0054334

**Published:** 2013-01-22

**Authors:** Quanjiao Chen, Shengping Huang, Jianjun Chen, Shaoqiong Zhang, Ze Chen

**Affiliations:** 1 State Key Laboratory of Virology, Center for Emerging Infectious Diseases, Wuhan Institute of Virology, Chinese Academy of Sciences, Wuhan, Hubei, China; 2 College of Life Sciences, Hunan Normal University, Changsha, Hunan, China; 3 Shanghai Institute of Biological Products, Shanghai, China; Johns Hopkins University - Bloomberg School of Public Health, United States of America

## Abstract

Two surface glycoproteins of influenza virus, haemagglutinin (HA) and neuraminidase (NA), play opposite roles in terms of their interaction with host sialic acid receptors. HA attaches to sialic acid on host cell surface receptors to initiate virus infection while NA removes these sialic acids to facilitate release of progeny virions. This functional opposition requires a balance. To explore what might happen when NA of an influenza virus was replaced by one from another isolate or subtype, in this study, we generated three recombinant influenza A viruses in the background of A/PR/8/34 (PR8) (H1N1) and with *NA* genes obtained respectively from the 2009 pandemic H1N1 virus, a highly pathogenic avian H5N1 virus, and a lowly pathogenic avian H9N2 virus. These recombinant viruses, rPR8-H1N1NA, rPR8-H5N1NA, and rPR8-H9N2NA, were shown to have similar growth kinetics in cells and pathogenicity in mice. However, much more rPR8-H5N1NA and PR8-wt virions were released from chicken erythrocytes than virions of rPR8-H1N1NA and rPR8-H9N2NA after 1 h. In addition, in MDCK cells, rPR8-H5N1NA and rPR8-H9N2NA infected a higher percentage of cells, and induced cell-cell fusion faster and more extensively than PR8-wt and rPR8-H1N1NA did in the early phase of infection. In conclusion, NA replacement in this study did not affect virus replication kinetics but had different effects on infection initiation, virus release and fusion of infected cells. These phenomena might be partially due to NA proteins’ different specificity to α2-3/2-6-sialylated carbohydrate chains, but the exact mechanism remains to be explored.

## Introduction

Influenza A viruses are single-stranded RNA viruses of the family *Orthomyxoviridae*, containing a segmented genome composed by eight RNA segments. Random combination of the 8 gene segments of influenza virus may produce novel viruses which exhibit different pathogenicity and propagation characteristics and even capacity for interspecies transmission [1], [2].

The spread of an influenza virus among humans is determined by interactions between human hosts and the virus. The 1918 flu pandemic was the most disastrous outbreak on record and caused more than 50 million deaths globally [3], but the virus did not reemerge afterwards and ultimately disappeared. The mortality rate of people suffering from highly pathogenic avian influenza virus infection was high [4],but person-to-person transmission of H5N1 virus was very rare and has been limited so far. The 2009 swine origin pandemic (S-H1N1) influenza virus caused mostly mild influenza-like illness, and the small percentage of severe cases occurred primarily among the young and the middle-aged [5]. Notably, this virus has displayed apparently higher transmissibility among humans than seasonal influenza viruses and H5N1 viruses have. Christophe et al. made an early assessment of transmissibility of S-H1N1 and case severity by analyzing the outbreak in Mexico, early data on international spread, and genetic diversity of the virus. They gave an estimated case fatality ratio of 0.4% (range: 0.3 to 1.8%) based on confirmed and suspected deaths reported by late April, 2009 [6]. These facts suggested that viruses of higher pathogenicity may not necessarily cause larger catastrophes for humans, while low pathogenic influenza viruses with easy human transmissibility could pose a serious public health threat. However, it is not yet possible to predict the extent of prevalence and severity of illness in humans caused by an influenza virus from its subtype, and the mechanism for virulence acquisition has not been well understood.

Hemagglutinin (HA) and neuraminidase (NA) are the two envelope glycoproteins on the surface of influenza virions. HA attaches to sialic acid on host cell surface to initiate virus infection [7]; NA removes sialic acid from cell receptor which HA binds to facilitate virus release [8]. As HA and NA recognize the same molecule (sialic acid) and have the opposite activity, sharp changes in either protein will affect virus replication [9]. But studies on what changes in HA-NA combinations affect biological properties of influenza viruses have been limited. In this study, using reverse genetics technology, we devised and produced three recombinant viruses in the background of PR8 (H1N1) virus which differ only in NA. The origins of three NA were diverse, and we set out to explore the properties of these recombinant viruses to evaluate the relative contribution of the NA protein.

The PR8 virus is a mouse-adapted, attenuated, laboratory H1N1 strain [10]. For the three NA origin viruses, the S-H1N1 virus is the virus that spread swiftly among humans in 208 countries in 2009 [11], and the H9N2 [12] and avian H5N1 virus [13] are respectively a low-pathogenic and a high-pathogenic strain isolated from chickens. These viruses are diverse but of field relevance and we hoped the study of different HA-NA combinations with the same HA could help to further the understanding of certain clinical, epidemiological and virological features of influenza viruses.

## Materials and Methods

### Plasmids, Viruses and Cells

Plasmids containing eight cDNAs of PR8 (pHW-PB2, pHW-PB1, pHW-PA, pHW-HA, pHW-NP, pHW-NA, pHW-M and pHW-NS) were constructed according to the method described by Hoffman [14]. Plasmids carrying *NA* gene segment from influenza virus A/H5N1 (A/chicken/Henan/12/2004, GenBank accession No. AY950246.1), A/H9N2 (A/Chicken/Jiangsu/7/2002, GenBank accession No. FJ384753.1) and swine A/H1N1 virus (*NA* was synthetized according to the nucleotide sequence of A/California/04/2009, GenBank accession No. ACP41107.1) were named pHW-H5N1-NA, pHW-H9N2-NA, pHW-H1N1-NA, respectively. The background virus used in this study, influenza virus A/PR/8/34 (H1N1) is a mouse-adapted strain. A/chicken/Henan/12/2004 (H5N1) and A/Chicken/Jiangsu/7/2002 (H9N2) influenza viruses were passaged and frozen at −80°C until use. The H5N1 virus was handled in a Biosafety Level 3 containment facility in the Wuhan Institute of Virology. 293T and MDCK cells were cultured in Minimal Essential Medium (MEM) containing 10% fetal calf serum (FCS).

### Generation of Recombinant Influenza Viruses

Recombinant virus was rescued as described previously [15], [16]. Briefly, 1 µg of each plasmid (pHW-PB2, -PB1, -PA, -HA, -NP, -M, -NS and pHW-H5-NA or pHW-H9-NA or pHW-SH1-NA) was combined with 18 µl transfection reagent Lipofectamine 2000 (2 µl per µg DNA, Invitrogen), incubated at room temperature for 30 min, and then transferred to monolayers of 10^6^ 293T cells on 6-well plates. Six hours later, the mixture was removed and replaced with Opti-MEM (Gibco-BRL) containing 0.3% BSA and 0.01% FCS. Forty-eight hours after transfection, culture medium was collected and inoculated onto 10-day-old SPF embryonated chicken eggs for virus propagation. Then allantoic fluids with positive HA titers were collected and stored at −80°C.

### Sodium Dodecyl Sulfate–polyacrylamide Gel Electrophoresis (SDS-PAGE)

The rescued viruses were inoculated onto 10-day-old SPF chicken embryos. Allantoic ﬂuid was collected 72 h later and centrifuged (5000×g) to remove cell debris. Then four viruses were inoculated in MDCK cells. At 72 h after infection, the supernatant were collected, first spun at 5000 g for 20 min for getting rid of cell debris and then ultracentrifuged at 4°C, 70,000×g for 3 h. Concentrated viruses were resuspended with SDS-PAGE sample loading buffer, incubated at 37°C for 30 min, and heated at 100°C for 1 min. The proteins were separated by 12% SDS-PAGE, and gels were stained with Coomassie brilliant blue G250.

### Replication Properties of Rescued Viruses and Titration of Viruses

Virus growth curve was used to analyze the replication properties of the rescued viruses. MDCK cell monolayers were inoculated with diluted virus at a MOI of 0.001, the inoculum was removed after incubation at 37°C for 1 h. The cells were washed and overlaid with 3 ml of MEM containing 1.0 µg/ml TPCK-trypsin. The supernatant was taken at 12 h, 24 h, 36 h, 48 h, 60 h, 72 h post infection.

The 50% tissue culture infectious dose (TCID_50_) was determined in MDCK cells which were incubated with 10-fold serially diluted viruses at 37°C for 72 h and cytopathic effect was observed. The 50% egg infectious dose (EID_50_) was determined in 10-day-old specific pathogen-free (SPF) embryonated chicken eggs which were incubated with 10-fold serially diluted viruses at 37°C for 48 h. The TCID_50_ and EID_50_ were calculated by Reed–Muench method [17]. The virus titer in each experimental group (n = 5) was represented by the mean ± SD.

### Virus Elution Assay

The ability of NA to elute virus bound on erythrocytes was assessed as described previously [18]. Briefly, fifty microliters of virus with HA titers of 1∶128 was incubated with 50 µl of 0.5% chicken erythrocytes at 4°C for 1 h. The mixture was then incubated at 37°C. In the next 8 hours, supernatant was taken periodically and measured for HA titer.

### NA Activity

The NA activities of the recombinant viruses were determined according to the method described by WHO manual [19]. In this assay the viral neuraminidase (an enzyme) acts on the substrate (fetuin) and releases sialic acid and the enzymatic reaction is stopped by adding arsenite reagent. Then the amount of sialic acid liberated is determined chemically with thiobarbituric acid which produces a pink color in proportion to free sialic acid. The color is then quantified with a spectrophotometer at wavelength 549 nm.

### Indirect Immunofluorescence Assay (IIFA)

MDCK cell monolayers were seeded on glass coverslips were inoculated with a virus solution which was removed after 1 hour of incubation, and the cells were incubated at 37°C for additional 3 h, 6 h or 12 h. At these specified time points, the cultures were fixed with 4% paraformaldehyde, permeabilized with 0.5% Triton X-100, blocked with 5% non-fat milk, and stained with polyclonal antisera to whole viruses. Next, the fluorescein isothiocyanate (FITC)-conjugated anti-mouse IgG (Millipore) secondary antibodies were added and then stained with Hoechst 33258 for 10 min. Fluorescent image analysis was performed on a Leica laser scanning confocal microscope with associated software as described previously [20]. Positive staining indicated successful virus entry in the cell [21].

### Flow Cytometry Analysis

Infected MDCK cells in suspension (2×10^6^) were incubated with PBS alone (mock) or anti-PR8 antibodies for 45 min on ice. Following extensive washing, the fluorescein isothiocyanate (FITC)-conjugated anti-mouse IgG (Millipore) secondary antibodies were added and incubated for 30 min on ice. After washing 3 times with PBS, the cells were fixed with 4% paraformaldehyde, and the number of infected cells was determined by flow cytometric analysis on an FACS-aria III flow cytometer (BD Biosciences).

### Pathogenicity and Lethality in BALB/c Mice

To test the pathogenicity of rescued viruses, BALB/c mice aged 6–8 weeks were anesthetized and inoculated intranasally (i.n.) with 20 µl of virus suspension at a titer of 1×10^6.5^ EID_50_. Bodyweight and survival of mice were recorded daily for 14 days after inoculation. To evaluate viral infection in the respiratory tract, three days after the challenge, five mice from each group were randomly taken for sample collection. The mice were anaesthetized with chloroform. The trachea and lungs were collected and washed three times by injecting with a total of 2 ml of PBS containing 0.1% BSA. The bronchoalveolar lavage was used for virus titration after removing cellular debris by centrifugation [13]. The animal experiment was approved by Animal Resource Center at the Wuhan Institute of Virology, Chinese Academy of Sciences (WIVA04201202).

### Statistics

The results of the test groups were evaluated by analysis of variation (ANOVA). The difference was considered significant if *p*-value was less than 0.05. The survival rates of the mice in the test and control groups were compared by using Fisher’s exact test.

## Results

### Generation of Recombinant Viruses Bearing Different NA Proteins

**Figure 1:**
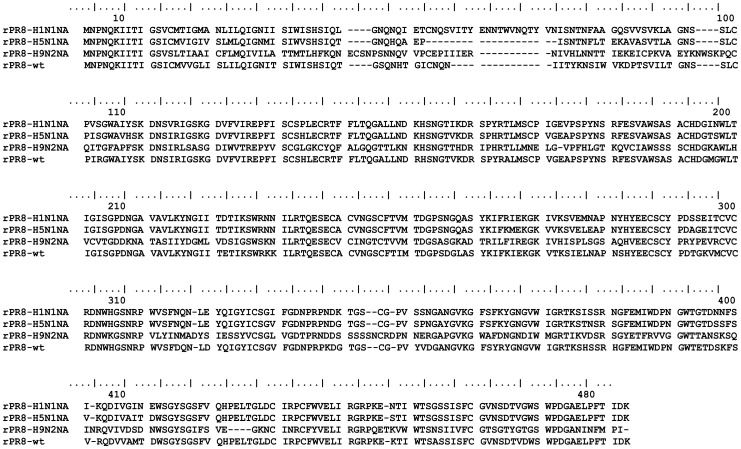
Alignment of the deduced amino acid sequences of the NA genes from the influenza virus strains A/Chicken/Jiangsu/7/2002(H9N2), A/California/04/2009 (H1N1), A/chicken/Henan/12/2004 (H5N1) and PR8 (H1N1).

Three recombinant influenza viruses were designed to share 7 gene segments with PR8 (H1N1) strain and have the *NA* gene segment from N1 subtype influenza viruses A/H5N1 and swine A/H1N1, or from the N2 subtype A/H9N2 virus, respectively. The NA proteins of H5N1, S-H1N1, H9N2 and PR8-H1N1 virus have 469, 449, 466 and 454 amino acids (aa), respectively and the sequences were aligned in Fig. 1. The NA amino acid homology between wild-type PR8 virus and H5N1, S-H1N1, or H9N2 was 71.4% 61.5% or 30.9%, respectively (Table 1).

**Table 1:** Virus strains generated by reverse genetics and the amino acids comparison of NA^a^.

Virus	NA Gene	Length	Homology^a^ (%)	Enzyme acitivity
rPR8-H5N1NA	H5N1	469	71.4	1∶10
rPR8-H9N2NA	H9N2	466	30.9	1∶10
rPR8-H1N1NA	Swine-H1N1	449	61.5	<1∶10
PR8-wt	PR8	454		1∶10

a The amino acids homology of wild-type PR8 virus and those of the H5N1, H9N2 or swine-H1N1.

The three viruses were successfully rescued by the eight-plasmid reverse genetics system, and they were named rPR8-H5N1NA, rPR8-H1N1NA, rPR8-H9N2NA.

The NA enzyme activity of the rescued viruses was evaluated and two viruses had activity comparable to that of PR8-wt, but the NA activity of rPR8-H1N1NA was slightly lower (Table 1).

### Characterization of the Recombinant Viruses

**Figure 2:**
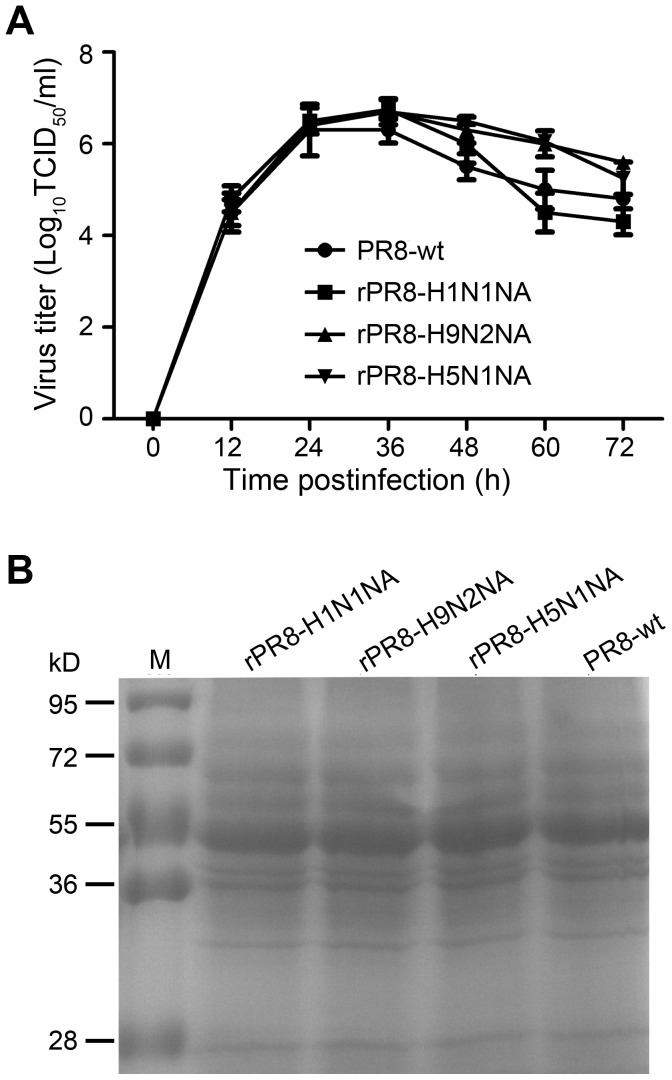
Characterization of recombinant influenza viruses. (A) Multiple-cycle growth curves of recombinant viruses on MDCK cells. MDCK cells were infected at a multiplicity of infection (MOI) of 0.001. The virus titers were measured by TCID_50_. The data shown are means ± SD for triplicate wells at each time point. Statistical significance (*p*>0.05). (B) SDS-PAGE analysis of expression of NA proteins in recombinant viruses. Concentrated viruses were resuspended with SDS-PAGE sample loading buffer, incubated at 37°C for 30 min, and heated at 100°C for 1 min. The protein was separated by 12% SDS-PAGE, gels were stained with coomassie brilliant blue G250.

The effects of *NA* replacement on virus infectivity were assessed by growth kinetics in both MDCK cells and SPF embryonated chicken eggs. MDCK cells were infected with the viruses at an MOI of 0.001 and the supernatants were collected at the indicated time and tested for TCID_50_. Results of replication kinetics showed that the titer of wild-type virus was 10^4.5^/ml at 12 h.p.i, rose to 10^6.3^/ml at 24 h.p.i and peaked (10^6.3^/ml) at 36 h.p.i. No significant difference was observed in the growth curves between the recombinant and wild-type viruses (Fig. 2A). Similarly, no difference was observed in the SPF chicken embryos infection experiment where all the viruses rapidly reached high yield of at least 10^8^ EID_50_ per ml (data not shown). In both tests, the recombinant viruses showed the same growth characteristics as those of the wild-type virus.

To examine the stability of the recombinant viruses, the viruses were subcultured on chicken embryos for 10 passages. They grew well and their HA titers were stable during the serial passage. RNA was extracted from the 10th-passage recombinant viruses for RT-PCR amplification of the *HA* and *NA* genes. No nucleotide sequence differences were detected between the 1^st^-passage viruses and 10^th^-passage viruses, demonstrating that these recombinant viruses were successfully generated and were genetically stable.

To examine if *NA* replacement would affect the ratio of NA protein in virions, the virions of the recombinant viruses were separated by 12% SDS-PAGE where equal loading of virions was ensured by an HA assay. The SDS-PAGE results showed that the proportion of NA protein in the recombinant virions were comparable to that of wild-type PR8 virus (Fig. 2B).

### Virulence in Mice

To compare the virulence and pathogenicity of recombinant viruses *in vivo*, BALB/c mice (n = 5 per group) were inoculated intranasally (i.n.) with 1×10^6.5^ EID_50_/20 µl of recombinant viruses or PR8-wt virus. The mortality, weight loss and viral titers in bronchoalveolar lavages were evaluated. The bronchoalveolar lavages were obtained at 3 and 6 days after infection.

**Figure 3:**
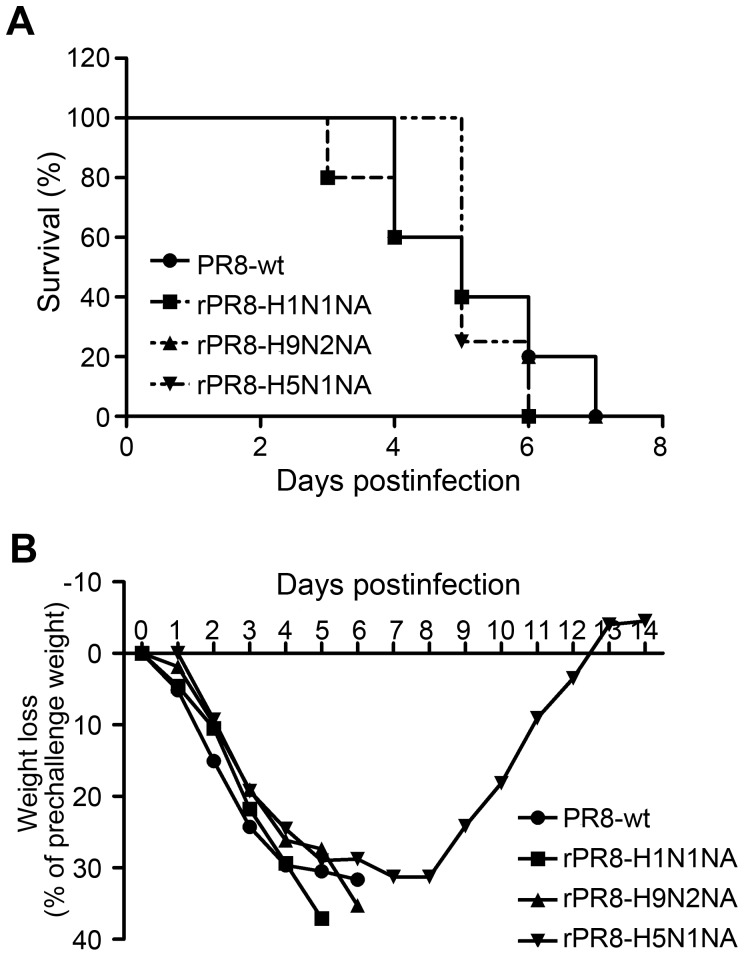
Pathogenicity of recombinant viruses in BALB/c mice. Survival rates (A) and bodyweight changes (B) after challenge with the viruses. BALB/c mice were intranasally inoculated with rPR8-H5N1NA, rPR8-H9N2NA, rPR8-H1N1NA or PR8-wt virus at 1×10^6.5^ EID_50_. The survival rates and bodyweights of five mice in each group were measured daily from the date of challenge to 14 days after challenge. Values represent mean ± SD of each group of mice.

As with PR8-wt virus, infection with the recombinant viruses caused serious clinical symptoms including piloerection, lethargy, anorexia, and bodyweight loss. The viruses caused fatal infection and all the infected mice died within 10 days of challenge except one in the rPR8-H5N1NA group (Fig. 3A).

High virus titers were detected in the bronchoalveolar lavages of mice on day 3 p.i. The residual lung virus titers of mice infected with PR8-wt virus was 10^7.3±0.5^/ml, the highest among the four viruses but not significant higher than those of mice infected with the recombinant viruses (*p*>0.05) (Table 2). The rate of bodyweight loss also did not differ significantly between mice infected with recombinant viruses or PR8-wt virus (Fig. 3B). The results indicated that the replacement of *NA* gene didn’t change the characteristics of the virus in terms of mouse-adaptation and lethality.

**Table 2:** Replication of recombinant viruses in mice^a^.

Virus	Virus titre (log_10_ EID_50_/ml, mean ± SD) in trachea lung wash	
	3 days (No.of survivors/no.tested)	6 days (No.of survivors/no.tested)
rPR8- H5N1NA	6.1±0.6 (5/5)	6.0±0 (2/5)
rPR8- H9N2NA	6.6±0.1 (5/5)	6.0±0.4 (2/5)
rPR8- H1N1NA	6.9±0.2 (5/5)	ND^b^ (0/5)
PR8-wt	7.3±0.5 (5/5)	6.0±0.4 (3/5)

a BALB/c mice were intranasally inoculated with recombinant or wild-type virus at 1×10^6.5^ EID_50_. On days 3 and 6 after infection, five mice from each group were killed for virus titration. Results are expressed as means ± SD.

b ND, Not done.

### Virus Elution in* vitro*

**Figure 4:**
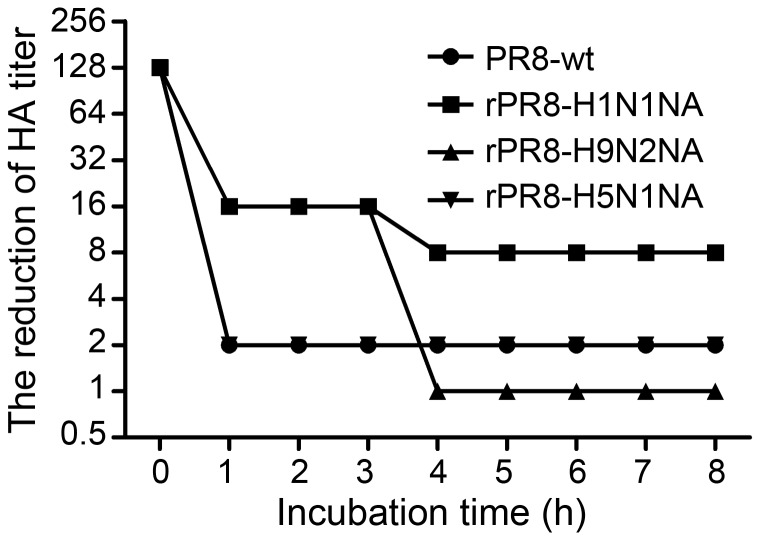
Virus elution in *vitro*. 50 µl two-fold dilutions of virus containing the HA titers of 1∶128 was incubated with 50 µl 0.5% chicken erythrocytes in microtiter plates at 4°C for 1 h. Then microtiter plates were incubated at 37°C, and the reduction of HA titers was measured periodically for 8 h.

To evaluate virus release rate *in vitro*, the four viruses, at a dose of 1∶128 by HA titer, were respectively first incubated with the same volume of 0.5% chicken erythrocytes at 4°C for 1 h and then incubated at 37°C for a prolonged time during which supernatants were taken periodically for HA assay which indicates the amount of virus released. After 1 h incubation at 37°C, the PR8-wt and rPR8-H5N1NA viruses showed a 64-fold reduction in HA titer and maintained at the level till the end of observation (8 h). The rPR8-H1N1NA virus showed an 8-fold reduction in HA titer after incubation 1 h at 37°C and again 2-fold reduction at 4 h till the end of experiment. rPR8-H9N2NA virus had an 8-fold reduction in HA titer after 1 h incubation at 37°C, which maintained for 3 h, and then its HA titer dropped further 16 fold; by the end of observation, its HA titer dropped by 128 fold (Fig. 4). As for the agglutination phenomenon, the cells incubated with PR8-wt and rPR8-H5N1NA presented agglutination clumping in the V-shaped 96-well microtiter plates from the start till the end (8 h). In contrast, in cells incubated with rPR8-H9N2NA and rPR8-H1N1NA, the agglutination phenomenon appeared only by 1 h after incubation at 37°C, where the supernatant of rPR8-H9N2NA and rPR8-H1N1NA were very clear and all the red blood cells have settled completely, and it stayed that way till the end of observation. Clearly, *NA* replacement dramatically altered the characteristics of virus elution from erythrocytes.

### The Initiation of Influenza Virus Infection

**Figure 5:**
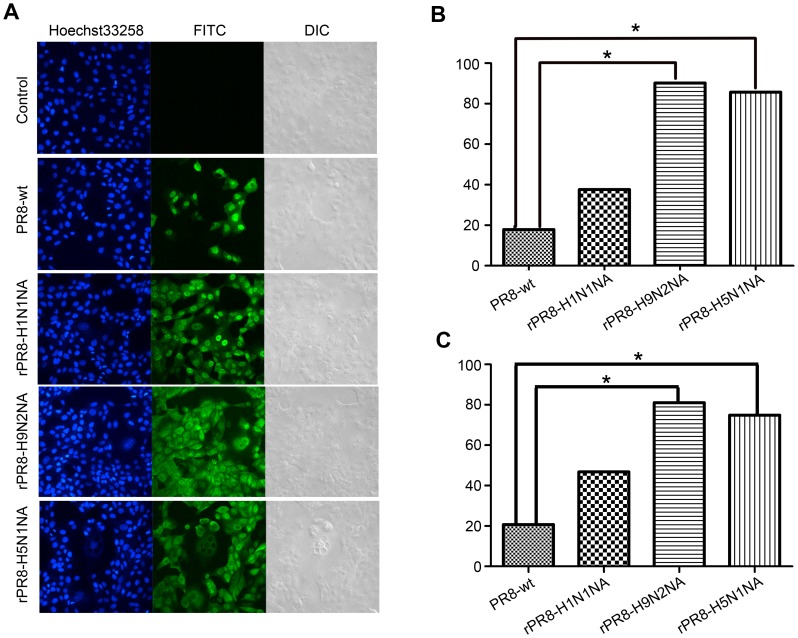
Impact of different NA on initiation of influenza virus infection. MDCK cells were infected at an MOI of 0.001 in the presence of 1 µg/ml TPCK-trypsin. After adsorption for 1 h at 37°C, the inocula were removed and the cultures were washed 3 times. The cells were incubated at 37°C for 6 h. At the indicated time, the cells were processed for immunofluorescence, and the infected cells were detected with polyclonal antisera to whole viruses. (A) Fluorescence images of the infected cells at 6 h p.i. Fluorescent photomicrographs showing the intracellular expression of virus protein in cell culture. The FITC-fluorescence signal was expressed as the infected cells. (B) Volocity Demo software analysis of the ratios of infected cells according to the Fig. 5A. *, statistical significance (*p*<0.05) (C) Flow-cytometric analysis of virus-infected cells at 6 h p.i. MDCK cells (2×10^6^) in suspension were incubated with PBS or anti-PR8 antibodies on ice. Then the FITC-conjugated IgG secondary antibodies were added. After washing, the cells were fixed and the number of infected cells was determined by flow cytometric analysis. *, statistical significance (*p*<0.05).

To determine if *NA* replacement affects the initiation of influenza virus infection, the number of MDCK cells infected by the virus 6 h.p.i was examined at a low MOI of 0.001 by flow-cytometric and by IIFA where positive FITC-fluorescence signal indicated infected cells. As shown in Fig. 5A, while no fluorescence could be detected in the uninfected cells, cells infected with PR8-wt and rPR8-H1N1NA showed 20.7% and 46.8% fluorescence positive, respectively. In comparison, cells infected with rPR8-H9N2NA and rPR8-H5N1NA showed significantly higher (*p*<0.05) positive rates (81.0% and 74.9%, respectively) (Fig. 5C). The percentage of FITC-fluorescence signal positive cells in Fig. 5B was calculated with the software Volocity Demo, and the trends in the four virus infected groups were consistent with those in Fig. 5C. This result suggested that NA replacement produced obvious effects on initiation of influenza virus infection. Furthermore, as seen from Fig. 5A, at 6 h after MDCK cells were infection by MOI of 0.001, the vRNPs of PR8-wt and rPR8-H1N1NA were seen only in the nucleus while vRNPs of rPR8-H9N2NA and rPR8-H5N1NA already exported into the cytoplasm. Therefore, a more rapidly infection process of rPR8-H9N2NA and rPR8-H5N1NA were observed than that of PR8-wt or rPR8-H1N1NA.

### Influenza Viruses Induced Cell-cell Fusion

**Figure 6:**
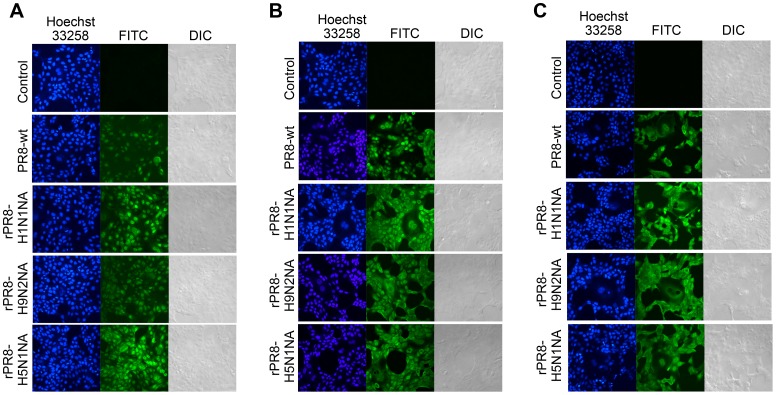
Influenza virus induced cell-cell fusion. MDCK cells were infected with the viruses at MOI of 0.1 or 0.001 in the presence of 1 µg/ml TPCK-trypsin. After adsorption for 1 h at 37°C, the inocula were removed and the cultures were washed 3 times. The cells were incubated for the indicated times at 37°C in the maintenance media. At the indicated time, the cells were processed for indirect immunofluorescence assay, and the infected cells were detected with polyclonal antisera to whole viruses. (A) MOI at 0.1, 3 h p.i. (B) MOI at 0.1, 6 h p.i. (C) MOI at 0.001, 12 h p.i.

MDCK cell monolayers were inoculated with viruses at a MOI of 0.001 or 0.1; at one hour post infection, the inoculum was removed and the cells were incubated at 37°C for 3 h, 6 h or 12 h and then the cells were fixed and stained. At MOI of 0.1, Cell-cell fusion was not obvious at 3 h for the cells infected with the four viruses. Cell volume enlargement was not obvious and there were only a few slightly larger cells. Cell nuclei were homogenous in size and there were no abnormally large ones, and there was no cytoplasmic fusion as observed by DIC (Fig. 6A). At 6 h post infection, merged cytoplasm and fusion of multiple cells were observed for all four virus groups, and cell volume of the rounded unfused cells was significantly smaller than the fusion cells (Fig. 6B). For PR8-wt infected group, the FITC field presented two dissolving nucleus appearing as holes, which could also be seen in the corresponding DIC image and the cytoplasm has already fused together. In cells infected with rPR8-H1N1NA, rPR8-H9N2NA and rPR8-H5N1NA, there were apparent cell enlargement or cytoplasm fusion, where the nucleuses gathered together or grew larger to about twice the size of neighboring cells. At MOI of 0.001 at 12 h post infection, the cells infected by four viruses all induced cell-cell fusion, fused cells were observed in large areas with clear cytoplasm and cell boundary. The vast majority of cells had huge volume (Fig. 6C).

The above results indicated that NA substitution could effectively change the cell fusion process, rPR8-H9N2NA and rPR8-H5N1NA viruses induced earlier and more obvious cell-cell fusion than PR8-wt or rPR8-H1N1NA virus at the same MOI, while at higher MOI cell fusion occurred earlier and more extensively.

## Discussion

HA, a type-I glycoprotein, plays a major role in virus replication in host cells. It attaches and fuses to cell surface via sialic acid and promotes virus entry by mediating membrane fusion between viruses and endosome [22]. NA, a type-II glycoprotein, can eliminate sialoglycoprotein from virus-infected cells and enable virus release [23]. Many studies have showed that low NA enzyme activity renders virus release from the virus-infected cells inefficient and leads to a large number of budding viruses gathered at the cell surface [24]; as the connection between these surface viruses and cell membrane is HA and sialic acid receptor of cell surface, a balance between HA and NA activities is crucial [25], [26]. In brief, HA activity should be high enough to ensure virus attachment to cells while the accompanying NA activity should not be too high so as to prevent HA binding to cells and not too low so as to be insufficient for releasing of progeny viruses [27].

In the current study, we obtained 3 recombinant viruses of the genetic background of PR8 (H1N1) which differed only in *NA*, which were from a H9N2, H5N1 and swine-H1N1 virus, respectively. Several studies have classified the NA subtypes into two groups: the first group consisting of N1, N4, N5, and N8 subtypes and the second group consisting of N2, N3, N6, N7 and N9 subtypes [28], whereas intra-group homologies are much greater than inter-group homologies [29]. The overall structure of NA was regarded as conserved among different subtypes of influenza A viruses, despite sequence homology could be as low as 30% [30], [31].

In the current study, successful rescue of the three recombinant viruses illustrated that the HA and NA activities of the viruses were by and large in functional balance. A series of tests were performed to examine the effect of NA replacement. The NA enzyme activity as assayed *in vitro* was equivalent among PR8-wt, rPR8-H5N1NA and rPR8-H9N2NA viruses, and was lower in rPR8-H1N1NA. These three recombinant viruses did not differ from the wild-type virus in growth kinetics in MDCK cells as measured by viral titer (TCID_50_) in cell culture medium collected at various time after infection. The proportion of NA incorporated into the virions also was not altered by *NA* replacement, as determined by SDS-PAGE. These recombinant viruses replicated efficiently in mouse lung without prior adaptation and caused death of mice in a manner comparable to that of the PR8-wt virus. However, for virus elution from chicken red blood cells, rPR8-H5N1NA and PR8-wt showed faster and significantly higher release than rPR8-H9N2NA and rPR8-H1N1NA did. Furthermore, in the early hours of infecting MDCK cells, rPR8-H9N2NA and rPR8-H5N1NA infected much more cells than rPR8-H1N1NA and PR8-wt viruses did in the early 3 h. These viruses induced fusion in infected MDCK cells, and, consistent with the infection initiation data, the fusion phenomenon occurred earlier and more extensive in rPR8-H9N2NA and rPR8-H5N1NA infected cells than in rPR8-H1N1NA or PR8-wt infected cells.

From these results, we could preliminarily conclude that *NA* replacement in the background of PR8 virus has little effect on virion composition, virus replication kinetics and infectivity and virulence in mice, but it has significant effect on virus elution from erythrocytes, and on efficiency of infection initiation and cell-cell fusion.

We do not yet have a unified explanation for the different observations made in the current study. There are certainly many factors to be taken into consideration. First, the species factor should be considered. The PR8-wt virus is a laboratory H1N1 strain which had been adapted in mouse. The H5N1 and H9N2 strains are field strains isolated from chickens. The S-H1N1 strain is also a field strain isolated from humans but is believed to be of swine origin. The cell line MDCK used is a canine kidney epithelium cell line, and the erythrocytes used in the elution study were from chickens. It is known that substrate specificity of NA protein is related to the species as well as the year the influenza virus is isolated from [32]. NA, neuraminidase, is an exosialidase which cleaves α-ketosidic linkage between the sialic (N-acetylneuraminic) acid and an adjacent sugar residue [33]. NA has substrates specificity in that it can discriminate between sialic acids and linkage type with the next residue (2–3, 2–6 or 2–8), as well as identify internal regions of the oligosaccharide chain. One example is that the key amino acid positions of NA’s neuraminic acid binding site are changed in viruses with α2-6-specificity (human, swine, and poultry H9N2 viruses) as compared to viruses whose HAs interact with α2-3-sialylated carbohydrate chains (i.e. avian and equine influenza viruses) [32]. Another example of species-specific NA substrate specificity is seen with data on N1 and N2 NAs of several duck, swine and human influenza virus isolates [32]. In these studies, all of the studied NAs desialylated α2-3-substrates better then α2-6 ones. For viruses with N1 neuraminidase, α2-3/α2-6 activity factor was ∼60 for duck viruses, ∼20 for swine viruses, and ∼4 for human viruses. For H9N2 influenza viruses, this α2-3/α2-6 relation for viruses isolated from poultry is in a range from 30 to 15, and for swine virus ∼6, finally, for human isolate ∼10. The data obtained in the current study might be explained in this light. The lower erythrocyte elution rate seen in Swine/human origin H1N1 and the avian origin H9N2 NA could be related to their source viruses’ α2-6-specificity and thus less efficient NA enzymatic cleavage and slower virus release.

But this α2-6-specificity could not explain why the two recombinant viruses with avian origin NA infected MDCK cells faster and more extensively than the two with mammal origin or adapted N1 subtype NA. Studies from Bovin’s group [32] have shown that NAs discriminate the fine structure of α2-3-substrates, that is, they discriminate between the structures of the inner parts of oligosaccharides. In addition, viral NA has been demonstrated by direct experimental evidence to play an essential role at the early stage of virus infection of human epithelium and NA is thought to promote virus entry [21], [34]. The experimental data of Flint et al. indicated that influenza virus HA protein could facilitate cell-cell fusion [35].

Variation in other functional domains, the enzyme active site, the stalk length, the sialic acid binding site and potential glycosylation sites [36], might also affect NA activity. Unlike serial mutations based on a single NA sequence, the three NAs in the current study are from diverse field strains of recent years and they differ in many sites, therefore it is not easy or too preliminary to speculate on possible mechanisms. Therefore, at this point we do not intent to give a full mechanism to explain the differential results observed, instead, we present the NA sequences and *in vitro* and *in vivo* data here as materials for future interpretation by the research community when more relevant data are accumulated.
